# Cytokine-Based Insights into Bloodstream Infections and Bacterial Gram Typing in ICU COVID-19 Patients

**DOI:** 10.3390/metabo15030204

**Published:** 2025-03-16

**Authors:** Rúben Araújo, Luís Ramalhete, Cristiana P. Von Rekowski, Tiago A. H. Fonseca, Cecília R. C. Calado, Luís Bento

**Affiliations:** 1 NMS—NOVA Medical School, FCM—Faculdade de Ciências Médicas, Universidade NOVA de Lisboa, Campo dos Mártires da Pátria 130, 1169-056 Lisbon, Portugal; rubenalexandredinisaraujo@gmail.com (R.A.);; 2 CHRC—Comprehensive Health Research Centre, Universidade NOVA de Lisboa, 1150-082 Lisbon, Portugal; 3 ISEL—Instituto Superior de Engenharia de Lisboa, Instituto Politécnico de Lisboa, Rua Conselheiro Emídio Navarro 1, 1959-007 Lisbon, Portugal; 4 IPST—Instituto Português do Sangue e da Transplantação, Alameda das Linhas de Torres 117, 1769-001 Lisbon, Portugal; 5 iNOVA4Health—Advancing Precision Medicine, RG11: Reno-Vascular Diseases Group, NMS—NOVA Medical School, FCM—Faculdade de Ciências Médicas, Universidade NOVA de Lisboa, 1169-056 Lisbon, Portugal; 6 Institute for Bioengineering and Biosciences (iBB), The Associate Laboratory Institute for Health and Bioeconomy-i4HB, Instituto Superior Técnico (IST), Universidade de Lisboa (UL), Av. Rovisco Pais, 1049-001 Lisbon, Portugal; 7 Intensive Care Department, ULSSJ—Unidade Local de Saúde São José, Rua José António Serrano, 1150-199 Lisbon, Portugal; 8 Integrated Pathophysiological Mechanisms, CHRC—Comprehensive Health Research Centre, NMS—NOVA Medical School, FCM—Faculdade de Ciências Médicas, Universidade NOVA de Lisboa, Campo Mártires da Pátria 130, 1169-056 Lisbon, Portugal

**Keywords:** cytokine profiling, bloodstream infections, Gram typing, ICU diagnostics, COVID-19, machine learning

## Abstract

**Background:** Timely and accurate identification of bloodstream infections (BSIs) in intensive care unit (ICU) patients remains a key challenge, particularly in COVID-19 settings, where immune dysregulation can obscure early clinical signs. **Methods:** Cytokine profiling was evaluated to discriminate between ICU patients with and without BSIs, and, among those with confirmed BSIs, to further stratify bacterial infections by Gram type. Serum samples from 45 ICU COVID-19 patients were analyzed using a 21-cytokine panel, with feature selection applied to identify candidate markers. **Results:** A machine learning workflow identified key features, achieving robust performance metrics with AUC values up to 0.97 for BSI classification and 0.98 for Gram typing. **Conclusions:** In contrast to traditional approaches that focus on individual cytokines or simple ratios, the present analysis employed programmatically generated ratios between pro-inflammatory and anti-inflammatory cytokines, refined through feature selection. Although further validation in larger and more diverse cohorts is warranted, these findings underscore the potential of advanced cytokine-based diagnostics to enhance precision medicine in infection management.

## 1. Introduction

Infections in intensive care units (ICUs) represent a significant challenge due to their high morbidity and mortality rates [1]. Early identification of bloodstream infections (BSIs) is critical, as delayed diagnosis can lead to severe complications, including septic shock, organ failure, and fatalities [2]. The Coronavirus Disease 2019 (COVID-19) pandemic, caused by the severe acute respiratory syndrome coronavirus 2 (SARS-CoV-2), has further exacerbated these challenges, with infection-related deaths rising due to both direct viral complications and secondary infections. Studies have highlighted that healthcare-associated infections (HAI) in ICU settings have often been overlooked amidst the pandemic’s demands, potentially contributing to misdiagnoses and delayed treatments [3]. Moreover, the overlap between inflammatory responses in COVID-19 and other critical infections [4] complicates traditional diagnostic approaches, emphasizing the need for more reliable biomarkers and predictive tools that are not used in isolation but rather in conjunction with thorough clinical evaluations and a comprehensive understanding of their biology, interferences, strengths, and limitations [5].

Traditional biomarkers, such as C-reactive protein (CRP), procalcitonin (PCT), and white blood cell (WBC) count, are widely employed in clinical practice to diagnose infections and assess their severity [6]. While these biomarkers are considered standard diagnostic tools, they are not without significant limitations. Culture-based techniques, which often accompany these markers, require extended timeframes to yield results and are influenced by the quality and quantity of the specimen, leading to potential delays in treatment decisions for critically ill patients [7]. Similarly, molecular assays, such as polymerase chain reaction (PCR)-based tests, offer faster results but are prone to false positives, which can result in unnecessary treatments and increased antibiotic resistance [8]. Furthermore, biomarkers like CRP and PCT often lack sensitivity and specificity in differentiating complex conditions, such as sepsis versus systemic inflammatory response syndrome (SIRS), or between ventilator-associated tracheobronchitis and pneumonia [9,10,11]. These limitations underscore the urgent need for more precise, rapid, and minimally invasive biomarkers to improve diagnostic accuracy in critical care settings [12]. Additionally, these diagnostic methods require significant time and specialized personnel, which can be costly in both financial and human resource terms.

Cytokines, small signaling proteins involved in immune responses, have emerged as potential biomarkers due to their ability to reflect the body’s inflammatory and immune status. Key cytokines such as interleukin-6 (IL-6) [13], interleukin-8 (IL-8) [14], and interleukin-10 (IL-10) [15] are frequently studied for their roles in inflammation and infection.

The balance between pro-inflammatory and anti-inflammatory cytokines is a critical determinant of clinical outcomes in ICU patients, including those with COVID-19. Pro-inflammatory cytokines, such as IL-6, Interleukin-1 beta (IL-1β), Tumor Necrosis Factor-alpha (TNF-α), and Interferon-gamma (IFN-γ), are vital for immune defense but, when dysregulated, can trigger a cytokine storm, exacerbating tissue damage, acute respiratory distress syndrome (ARDS), and multi-organ failure [16]. Conversely, anti-inflammatory cytokines like IL-10 and interleukin-13 (IL-13) mitigate excessive immune activation, yet their imbalance often worsens disease severity [17]. In severe COVID-19 infections, elevated IL-10 levels may reflect a compensatory but inadequate response to hyperinflammation, dominated by IL-6, TNF-α, and IL-1β. These dynamics highlight the need for therapies that balance immune modulation to prevent hyperinflammation without compromising pathogen-specific responses, particularly in ICU settings where precision interventions can improve outcomes [18,19].

Despite their potential, cytokines as biomarkers face limitations that constrain their clinical utility. Many studies report widely varying diagnostic performance metrics, with AUC values ranging from 0.71 to 0.83 when using IL-6 alone [20]. Research often focuses on elevated cytokine levels to differentiate between types of infections, rather than solely relying on diagnostic accuracy [21]. Furthermore, research tends to prioritize a limited subset of inflammation-related cytokines, such as IL-6, IL-8, and TNF-α, which are associated with generalized immune responses rather than specific infection states [22,23]. This narrow scope neglects the complexity of cytokine interactions and their capacity to reflect dynamic physiological conditions. While cytokine ratios have shown potential for enhancing diagnostic precision, their application remains underexplored [24].

Researchers have explored the utility of cytokines and their ratios for diagnosing BSIs and discriminating between bacterial Gram types. Several studies have focused on cytokine profiles for detecting bacteremia. For instance, a high IL-10/TNF-α ratio measured on Day 1 was found to be predictive of persistent bacteremia, while values recorded on Day 4 could predict 30-day mortality [25]. Elevated levels of IL-6, IL-8, and Interferon gamma-induced protein 10 (IP-10, also known as CXCL10) were associated with bacterial infections in febrile patients, irrespective of culture positivity, highlighting their potential as robust biomarkers [26]. In BSIs caused by *Klebsiella pneumoniae*, persistent IL-6 and IL-10 elevation correlated with increased mortality, while high arginase levels were linked to carbapenem-resistant strains, with AUCs of 0.726 for arginase and 0.805 for its combination with TNF-α and Interleukin-4 (IL-4) [27]. Additionally, cytokines such as Interleukin-2 (IL-2), IL-6, IL-10, and Interleukin-17A (IL-17A) were significantly elevated in patients with bloodstream Candida infections compared to bacterial BSIs, demonstrating their capacity to differentiate between fungal and bacterial pathogens [28].

In addition to bacteremia, cytokines and their ratios have been investigated for use in stratifying bacterial Gram types. In one study, it was highlighted that IL-6 and IL-10 levels appeared elevated while IL-2 levels were decreased in Gram-negative infections, with AUCs for IL-2 and IL-10 of 0.581 and 0.661, respectively, whereas a combined logistic regression model for IL-2 and IL-10 achieved an AUC of 0.735 for distinguishing Gram-negative from Gram-positive infections [29]. Others reported a promising IL-6/IL-10 ratio (AUC = 0.822) as a rapid diagnostic tool for Gram-negative and Gram-positive bacteremia [30]. Despite extensive research efforts in this field, most studies still rely on conventional cytokines or predefined ratios, limiting the scope for discovering novel biomarkers or combinations that could significantly enhance diagnostic accuracy [31].

Selecting an appropriate biofluid is critical for ensuring reproducibility and reliability in cytokine biomarker studies. In this study, serum was chosen for its stability and suitability for long-term storage. Derived from whole blood, serum lacks the clotting factors that could introduce variability, providing a consistent medium for analyzing immune responses [32,33]. Its utility has been demonstrated in studies on inflammation [34], sepsis [35], and BSIs [28].

This study addresses the diagnostic challenges of BSIs in ICU COVID-19 patients by leveraging cytokine profiling and machine learning methodologies. Using serum samples from 45 ICU patients, we investigated the potential of a 21-cytokine assay to differentiate between patients without BSIs (23 patients) and those with confirmed BSIs (22 patients), where bacterial infections were further stratified by Gram type. In addition to evaluating the traditional cytokine levels and ratios reported in the literature, this study aimed to identify potentially more effective ratios for improving diagnostic precision. These findings underscore the potential of cytokine profiling to improve clinical decision-making in critical care.

## 2. Materials and Methods

### 2.1. Workflow Overview

**Figure 1:**
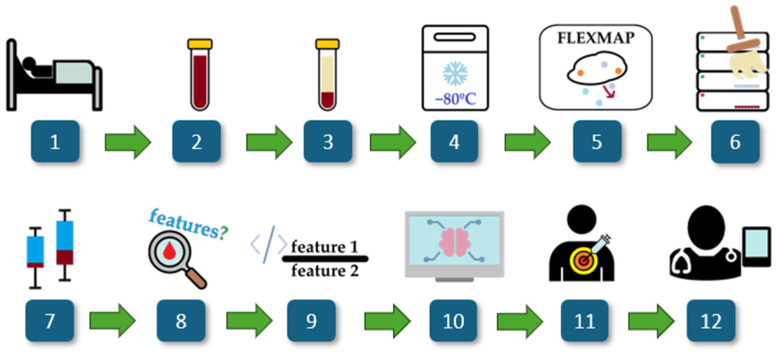
Overview of the study. The workflow consists of: (1) ICU COVID-19 patient selection, (2) blood collection, (3) serum extraction, (4) storage at −80 °C, (5) cytokine profiling via a multiplex assay, (6) data processing and cleaning, (7) statistical analysis, (8) univariate and multivariate cytokine analysis, (9) computational generation of cytokine ratios, (10) feature selection using FCBF, (11) machine learning modeling for biomarker discovery, and (12) clinical interpretability assessment.

Figure 1 provides a schematic representation of the study workflow, detailing the key steps from patient selection to model evaluation. The process begins with the recruitment of ICU patients diagnosed with COVID-19, followed by standard blood collection and serum extraction. Serum samples are then preserved at −80 °C to maintain stability for cytokine profiling. A high-sensitivity multiplex assay is utilized to quantify a panel of 21 cytokines, with the data subsequently processed through extraction, transformation, and cleaning pipelines. Statistical analyses and computational methods, including univariate and multivariate assessments, guide the selection of cytokine ratios. Feature selection algorithms then identify the most relevant potential biomarkers, which are then used to train machine learning models for distinguishing bloodstream infections and Gram typing.

### 2.2. Study Population

Forty-five patients who were admitted to the ICU of Hospital São José in Lisbon, a tertiary hospital, were included in this study, part of the Predictive Models of COVID-19 Outcomes for Higher-risk Patients Towards a Precision Medicine (PREMO) project and approved by the hospital’s Ethics Committee. Informed consent was obtained from all patients or from their immediate family members for data collection. All demographic, clinical, and laboratory data were collected from the hospital’s electronic medical records system and anonymized.

All patients included in this study were critically ill COVID-19 patients, with infection confirmed via real-time polymerase chain reaction tests (RT-PCR). Biological samples were collected from patients admitted to the ICU between 12 November 2020, and 24 September 2021. The final dataset comprises samples from patients without bloodstream infections (no BSIs, *n* = 23) and patients with confirmed bloodstream infections (presence of BSIs, *n* = 22), of which 7 were Gram-negative and 15 were Gram-positive. BSIs were identified using standard microbiology techniques, with hemoculture testing performed on blood samples collected directly from a peripheral vein and analyzed by the hospital laboratory. Gram type was determined from bacterial isolates using routine microbiological identification tests. The biological samples used for cytokine profiling were collected on the same day, ensuring that only patients with confirmed BSIs concurrent with serum collection were included in the study.

### 2.3. Collection of Biological Samples

Peripheral blood was collected in VACUETTE^®^ tubes (Greiner Bio-One GmbH, Kremsmünster, Austria) without anticoagulant, using standard blood collection procedures. Serum was obtained by centrifuging the samples at 3500 rpm for 10 min (Mikro 220T, Andreas Hettich GmbH & Co. KG, Tuttlingen, Germany) and stored at −80 °C until cytokine profiling.

### 2.4. Cytokine Profiling

Serum cytokines were analyzed, using a MILLIPLEX MAP 384-well Human High-sensitivity T Cell Panel immunology multiplex assay, to profile a comprehensive set of 21 cytokines (HSTC384-28K, Millipore, Merck, Germany): Interferon-inducible T cell alpha chemoattractant (ITAC), granulocyte-macrophage colony-stimulating factor (GM-CSF), Fractalkine (also known as CX3CL1), IFN-γ, IL-10, macrophage inflammatory protein-3 alpha (MIP-3α), Interleukin-12p70 (IL-12p70), IL-13, IL-17A, IL-1β, IL-2, Interleukin-21 (IL-21), IL-4, Interleukin-23 (IL-23), Interleukin-5 (IL-5), IL-6, Interleukin-7 (IL-7), IL-8, macrophage inflammatory protein-1 alpha (MIP-1α), macrophage inflammatory protein-1 beta (MIP-1β), and TNF-α. The assay was performed according to the manufacturer’s protocol, using a FLEXMAP^®^ 3D system with xPONENT^®^ software version 4.3 for data acquisition and analysis (Luminex Corporation, Austin, TX, USA).

### 2.5. Data and Statistical Analysis

The demographic variables in this study included age and gender [36,37], while clinical characteristics comprised body mass index (BMI) [38] and the presence of comorbidities [39]. The types of respiratory support provided, specifically, invasive mechanical ventilation (IMV) [40] and extracorporeal membrane oxygenation (ECMO) [41], were also considered. These variables were selected for their clinical relevance in COVID-19 patients, as they are significant prognostic factors for ICU outcomes. It was assessed whether these variables exhibited statistically significant differences between the two main patient groups: patients with confirmed BSIs and those without BSIs, as this allows the effect of confounding variables to be minimized. Statistical significance for this particular set of variables was defined as a two-sided *p*-value of less than 0.01.

Statistical analysis of the patients’ demographic and clinical characteristics was conducted using Student’s *t*-test for continuous variables with a normal distribution and the Mann–Whitney *U* test for non-normally distributed continuous variables. For categorical data, chi-square (*χ*^2^) and Fisher’s exact tests were applied, with Fisher’s test specifically used for smaller sample sizes. Continuous variables were represented as medians and interquartile ranges (25th to 75th percentiles), while categorical data were presented as absolute frequencies and percentages. All statistical analyses were performed using IBM SPSS Statistics software, version 27 (IBM Corp., Armonk, NY, USA).

The t-distributed stochastic neighbor embedding (SNE) technique, an unsupervised classification method, was conducted to visualize patient groupings. t-SNE is a dimensionality reduction technique that projects high-dimensional data into two or three dimensions while preserving relative distances between the data points, revealing clusters or groupings in complex datasets [42]. All t-SNE visualizations were pre-processed, with the ‘Normalize data’ option enabled to reduce potential bias from differences in feature scales. The perplexity setting was adjusted to ‘preserve global structure’, which aims to retain the relative positioning of groups within the dataset, ensuring that larger patterns in the data are better represented in the low-dimensional space. Additionally, supervised classification models—k-nearest neighbors (kNN), naïve Bayes, random forest, support vector machine (SVM), and decision tree—were developed. Five-fold cross-validation was implemented, in which the dataset was divided into five equal parts, with the model trained on four parts and tested on the remaining part in each iteration. This ensured that each sample was tested once. This approach provides a more robust estimate of model performance and mitigates the risk of overfitting by ensuring that no single subset of data disproportionately influences the results. By averaging model performance across all folds, cross-validation reduces model dependency on specific data distributions, helping to ensure that the reported performance metrics reflect the true predictive capability within a specific dataset.

Feature selection for cytokines and their ratios was performed using a fast correlation-based filter (FCBF), an entropy-based algorithm that ranks variables by their importance in discriminating target groups (e.g., patients with BSIs from those without BSIs, or Gram-negative from Gram-positive). FCBF assigns values to each feature within a range of [0,1], where 1 indicates a feature whose value completely predicts the target class, while 0 represents no predictive value [43]. Only features with a score greater than 0 were retained for further analysis, ensuring that only the most informative cytokine ratios were considered. Given the extensive number of cytokine-derived ratios generated in this study, FCBF was chosen for its ability to efficiently filter out redundant features, a challenge that alternative scoring methods may struggle with when applied to such high-dimensional datasets. Feature selection was applied to the entire dataset before cross-validation, as the primary goal was to identify biologically relevant cytokine ratios rather than solely optimize predictive performance [44]. While this approach may potentially carry a risk of data leakage—where information from the test set could inadvertently influence feature selection, leading to overly optimistic model performance—it ensures that selected features reflect global discriminative power rather than being dependent on a specific fold of the dataset. This does not invalidate the need for future studies that should strive to incorporate larger datasets with distinct subsets for training, testing, and validation to further enhance model stability and generalizability [45].

The analyses mentioned above were conducted using the Orange: Data Mining Toolbox, version 3.36.2 (Bioinformatics Lab, University of Ljubljana, Ljubljana, Slovenia) [46].

Nomograms [47] were applied to the datasets after FCBF for both target classes in this study. A nomogram is a graphical tool that illustrates the contribution of each feature to a predictive model, allowing for an intuitive interpretation of their impact on the target outcome. The log odds ratio scale was used to quantify the influence of each feature, as it is widely understood and preferred in clinical contexts for comparing the relative effects of variables. Features were displayed in ranked order, reflecting their importance as determined by the FCBF method, ensuring that the most relevant features appeared prominently for interpretation. The blue markers on the nomogram serve as reference points for how the different ranges contribute to classification.

To complement the cytokine assay of 21 cytokines from the MILLIPLEX MAP 384-well Human High-sensitivity T Cell Panel, all possible ratio combinations were programmatically calculated [48] using a maximum of three cytokines (as a sum) in both the numerator and denominator. Pro-inflammatory cytokines (ITAC, GM-CSF, Fractalkine, IFN-γ, IL-17a, IL-1β, IL-2, IL-12p70, IL-23, IL-6, IL-8, MIP-1α, MIP-1β, and TNF-α) were assigned to the numerator, while anti-inflammatory cytokines (IL-10, IL-4, IL-13, IL-5, IL-7, and IL-21) were assigned to the denominator.

It is acknowledged, however, that classifying certain cytokines as strictly pro- or anti-inflammatory remains a subject of debate in the literature. For instance, IL-5, IL-7, and IL-21 are sometimes cited as predominantly pro-inflammatory [49,50,51], whereas other studies highlight immunomodulatory or anti-inflammatory roles for these same cytokines [52,53,54]. Similarly, IL-2 is widely regarded as a pro-inflammatory T cell growth factor [55], yet emerging evidence highlights its critical role in supporting regulatory T cell homeostasis and mediating anti-inflammatory effects [56]. Despite these nuances, the above categorization was adopted for the ratio-based analyses in the present study. Following this classification approach, the total number of theoretical ratios was reduced from over 1.5 million to 19,229 biologically relevant combinations. These ratios, along with the 21 original cytokine concentrations (for a total of 19,250 features), were then fed to FCBF, an entropy-based scoring method, to streamline the dataset and identify the features most strongly contributing to the discrimination of the target classes: the presence of BSIs and Gram type.

The analysis was further expanded to evaluate cytokine distributions and compare their levels across the patient groups (e.g., patients with BSIs vs. those without BSIs). Only outliers up to 1.5 times the interquartile range (IQR) were included in the displayed boxplots to avoid compression of the visualization scale. Depending on the normality of each group’s data, assessed via the Shapiro–Wilk test, either Student’s *t*-test (for normally distributed data) or the Mann–Whitney *U* test (for non-normal distributions) was used for statistical comparison, with univariate analysis allowing for individual comparisons of cytokines. Boxplots were generated to visualize group distributions, with the median, interquartile ranges, and potential outliers highlighted. Statistical significance was annotated directly on the plots, with *p*-values displayed.

## 3. Results and Discussion

### 3.1. Study Population Characteristics

The current study included 45 ICU patients from a tertiary hospital in Lisbon, of whom 22 had a confirmed BSI based on positive hemoculture results. The remaining patients had no detected BSI, as indicated by negative hemoculture results. The two patient groups (BSIs and No BSIs) did not differ statistically (*p* > 0.01) in terms of demographic characteristics (age, gender) or clinical variables such as ECMO, IMV, the presence of comorbidities, and BMI (Table 1). This minimizes the potential effects of confounding variables [48].

### 3.2. Commonly Reported Cytokines and Ratios

Cytokines, simple ratios, and traditional laboratory markers such as PCT and CRP have been widely explored as biomarkers. However, their application is often limited to specific contexts, with individual studies focusing on bloodstream discrimination, bacterial Gram typing, sepsis, disease severity, or mortality in isolation. Many of these studies examine the same limited cytokines and ratios in relatively small and specific populations. To establish a foundation for our analysis, we summarize these commonly reported variables in the following section, linking them to Table 2, where their behavior in our cohort is analyzed and compared to findings in the literature.

For hemoculture discrimination, multiple features have been explored as biomarkers. Studies have reported elevated CRP, PCT, IL-6, IL-10, WBC, neutrophil percentage (NE%), and erythrocyte sedimentation rates in BSI patients compared to those with local bacterial infections [57]. Cytokines such as IL-6, IL-8, and IP-10 were found to be significantly elevated in bacterial infections, regardless of culture positivity [26]. Furthermore, in a cohort of 51 patients with bloodstream Candida infections, compared to 20 with bacterial bloodstream infections, elevated IL-2, IL-17A, IL-6, and IL-10 levels were reported as potential discriminatory biomarkers [28]. However, the patient population was highly imbalanced and included a diverse set of ICU, oncology, and surgical patients, with no clear normalization for severity or comorbidities. Cytokine ratios have also been explored, with a high IL-10/TNF-α ratio on Day 1 predicting persistent bacteremia and correlating with 30-day mortality when measured on Day 4 [25]. However, most of these studies either combine BSI detection with mortality prediction, lack focus on bacteremia alone or are limited to highly specific cohorts such as extremely low birth weight infants, where elevated Th1 cytokines (e.g., IFN-γ) and IL-10 were linked to increased infection risk [58].

For Gram typing, elevated IL-6 and IL-10 levels have been associated with Gram-negative infections, with the IL-6/IL-10 ratio achieving an AUC of 0.822 for distinguishing Gram-negative from Gram-positive bacterial infections [30]. The IL-2/IL-10 ratio also demonstrated a capacity to differentiate Gram-negative from Gram-positive infections with an AUC of 0.735 in a study on sepsis and intracranial infections [29].

Cytokines have also been investigated for other clinical applications beyond hemoculture and Gram typing. Some studies have explored their use as biomarkers for defining clinical cutoff values, with cytokines such as IL-2, IL-6, IL-10, and TNF-α being proposed as reference points for inflammatory conditions [59]. Additionally, cytokines like IL-6 and IL-10 have been evaluated for their association with infection severity, including severe adenovirus infections where elevated levels of these cytokines correlated with more severe cases [60]. In sepsis, cytokine modulation has also been explored as a therapeutic strategy, with IL-6, IL-10, and TNF-α serving as targets for managing hypercytokinemia in both sepsis and COVID-19 patients [61].

To provide a comprehensive overview, Table 2 compiles all the cytokines, simple ratios, and traditional laboratory or clinical markers commonly found in the relevant literature (or the closest equivalent in our cohort data, e.g., lymphocyte count in place of white blood cell count), using values recorded from our own cohort. These values will be used to determine whether the levels, ratios, or markers were elevated or decreased per group, with their statistical significance being reported accordingly. This approach aims to ensure transparency and thoroughness by replicating previously reported biomarkers while expanding the scope of the analysis.

The results obtained in the present cohort (Table 2) demonstrate both convergence and divergence when compared with previously reported biomarkers for BSI discrimination and Gram typing, as outlined at the beginning of this section. The specific areas of agreement and divergence are detailed below.

**BSIs: Presence vs. Absence**

Several cytokines and traditional laboratory markers that were previously reported as biomarkers for BSI discrimination, such as CRP, PCT, IL-6, and IL-10, were included in the present analysis. Significant elevation of IL-6 (*p* = 0.020) and the IL-6/IL-10 ratio (*p* < 0.001) was observed in patients with BSIs, aligning with the literature indicating IL-6’s role as a marker of systemic inflammation and BSI presence [26,57]. However, CRP and PCT, commonly cited as elevated in BSI [57], were not statistically different between patients with and without BSIs (*p* > 0.05).

Cytokines IL-10, IL-8, and TNF-α showed no significant differences between BSI and non-BSI patients, despite being reported as elevated in BSIs in prior studies [26,28]. This divergence may be partly explained by the substantial proportion of missing values for traditional laboratory markers in the dataset (12.5% for BSI and 82.6% for non-BSI), which could be influenced by the characteristics of the cohort, including the presence of COVID-19 cases.

Non-significant differences were also observed for IL-2 and IL-17A, despite previous research highlighting their elevation in BSIs caused by Candida species [28]. However, differences in patient populations (the present cohort did not include Candida infections), along with the small group sizes, may have influenced these findings.

**Gram-Positive vs. Gram-Negative**

The analysis revealed statistically significant differences in CRP (*p* = 0.006) and lymphocytes (*p* = 0.014) between Gram-positive and Gram-negative infections. IL-17A levels were observed to be higher in Gram-positive infections, although the difference did not reach statistical significance (*p* = 0.056). Consistent with the previous literature, higher IL-6 and IL-10 values and a higher IL-6/IL-10 ratio were observed in Gram-positive infections compared to Gram-negative cases, supporting reports where the IL-6/IL-10 ratio has been shown to distinguish Gram typing with an AUC of 0.822 [30]. However, the IL-2/IL-10 ratio did not reach statistical significance in the present cohort (*p* = 0.758), contrasting with its previously reported discriminatory capacity [29].

Interestingly, while both CRP and IL-6 were significantly higher in Gram-positive infections (CRP: 224.85 vs. 118.40 mg/dL; IL-6: 21.55 vs. 3.97 pg/mL), PCT did not show a statistically significant difference (PCT: 0.35 vs. 0.66 ng/mL; *p* = 0.953), diverging from prior reports that emphasized its elevation in Gram-negative infections [30]. This divergence may be influenced by the lower number of Gram-negative cases and the proportion of missing data in this subgroup (28.6% for Gram-negative and 5% for Gram-positive).

**Convergence With and Divergence From the Literature**

The findings support previously established results, particularly the elevation of IL-6 and IL-6/IL-10 ratios in both patients with BSI and Gram-positive infections. However, the lack of statistically significant differences for other markers, such as PCT and IL-10, contrasts with earlier reports, emphasizing the variability that may arise due to cohort size, infection type diversity, missing data, and the presence of COVID-19 infection. The limited number of BSI and non-BSI cases in the present cohort may have influenced the statistical power to detect differences. Therefore, while the data confirms the relevance of IL-6 and IL-6/IL-10 ratios as key markers for both BSI detection and Gram typing, further studies with larger, more balanced cohorts are needed to clarify the roles of other cytokines such as PCT, IL-10, and the IL-2/IL-10 ratios.

Additionally, the reported missing values regarding the traditional laboratory markers must be considered when interpreting these results (Table 2). While some studies may choose to apply statistical imputation methods to estimate missing values (e.g., mean, median, or multiple imputation), the present dataset was retained in its original structure to ensure transparency and prevent the introduction of artificial bias. While this limitation is inherent in real-world research, even more so when focused on ICU environments, future studies with expanded datasets and more comprehensive biomarker collection could potentially mitigate this issue.

In the next section, a univariate cytokine analysis for the complete multiplex assay is presented regarding both target classes: BSI presence and Gram typing.

### 3.3. Univariate Cytokines Analysis

In this section, the median and IQR values for all 21 cytokines are presented for BSI versus non-BSI patients and for Gram-negative versus Gram-positive patients. Additionally, absolute differences, percentage differences (% difference), and fold changes were calculated to highlight disparities between the groups. To ensure clinical relevance, BSI patients are used as the reference for BSI analysis, and Gram-negative patients are used as the reference for Gram typing. The absolute difference reflects the raw magnitude of change between groups, while the % difference incorporates directionality, indicating whether cytokine levels are relatively higher or lower in the reference group. Fold changes further complement these analyses by showing proportional differences; values of greater than 1 suggest elevated cytokine levels in the reference group, while values of less than 1 indicate lower levels. These metrics, alongside *p*-values for statistical significance, provide a comprehensive view of cytokine behavior in the context of BSIs and Gram typing in the present cohort.

#### 3.3.1. Bloodstream Infections

**Figure 2:**
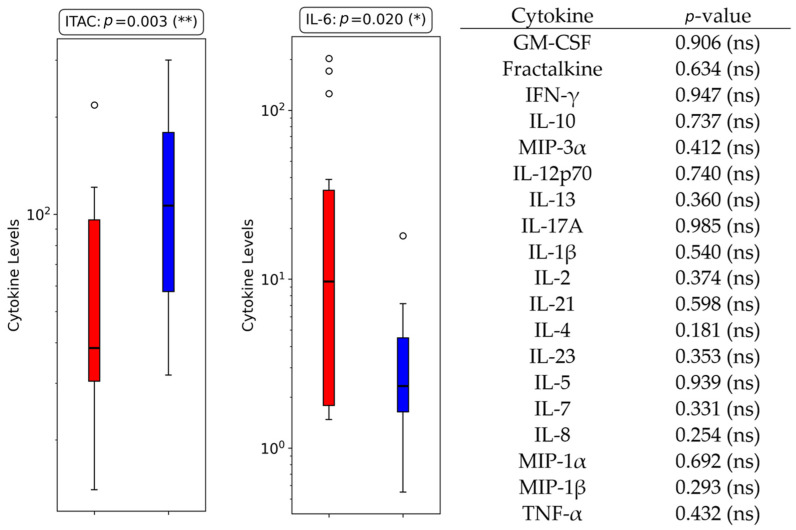
Boxplots displaying cytokine levels for the two cytokines with statistically significant differences between BSI and non-BSI groups in ICU COVID-19 patients. All the other cytokines analyzed were non-significant, with their respective *p*-values listed in the right panel for reference (ns = non-significant; *: *p* < 0.05; **: *p* < 0.01).

The analysis of cytokine levels between BSI and non-BSI patients revealed notable differences in cytokine expression. Among the 21 cytokines analyzed, statistically significant differences were observed for 2 cytokines (ITAC and IL-6), with *p*-values of 0.003 and 0.020, respectively (Figure 2, Table 3). ITAC levels were significantly lower in the BSI group compared to the non-BSI group (median: 60 vs. 106), with an absolute difference of 47, a percentage difference of −79%, and a fold change of 0.56.

Similarly, IL-6 levels were higher in the BSI group (median: 21.50 vs. 2.78), with an absolute difference of 18.71, a percentage difference of 672.65%, and a fold change of 7.73.

IL-6’s elevation in BSI patients aligns with its well-documented role as a biomarker of systemic inflammation. Studies have highlighted IL-6 as a critical mediator in both bacterial and viral infections, particularly for its involvement in driving acute-phase responses and as a marker of disease severity [62,63]. Elevated IL-6 levels have been reported in febrile infections and bacteremia, reflecting the severity of systemic inflammation and aiding in the differentiation of infection types. In critically ill patients, IL-6 concentrations correlate with worse 90-day survival, organ dysfunction, and the need for organ support therapies such as vasopressors or renal replacement therapy [64]. Additionally, IL-6 has been proposed as a valuable indicator for early prognosis and follow-up in septic shock patients [65] and has demonstrated utility in conjunction with clinical scoring systems like SOFA for predicting 28-day mortality [66].

Conversely, the pronounced reduction in ITAC levels in the BSI group may reflect differences in immune responses to bloodstream infections. ITAC plays a role in the chemotaxis of T cells and NK cells, and its decreased levels in BSI patients might be linked to pathogen-specific immune suppression or systemic depletion during bacterial infections. This is supported by studies indicating distinct cytokine profiles in bacterial and viral infections, where ITAC levels are more prominently elevated in viral infections [67]. The reduction in ITAC in BSI patients could reflect a bacterial-specific immune signature or the modulation of chemokine production during bacteremia [68].

While most other cytokines did not show statistical significance, several trends were observed. For instance, MIP-1α and IL-8 exhibited percentage differences of 25.91% and 73.52%, respectively, with fold changes of 1.26 and 1.74, suggesting higher levels in the BSI group. IL-8, in particular, has been associated with neutrophil activation and inflammatory responses during bacterial infections. Its increased levels in BSI patients (median: 16.74 in BSI vs. 9.65 pg/mL in non-BSI patients; % difference: 73.52%) further support its role in neutrophil-driven inflammation and systemic cascades in bloodstream infections [69].

The near-equilibrium seen in the patient distribution between BSI (*n* = 22) and non-BSI (*n* = 23) minimizes the likelihood of sample size bias influencing the observed differences. However, variations in treatment regimens or baseline comorbidities may still account for some discrepancies. Overall, these results highlight the potential of IL-6 and ITAC as biomarkers for systemic inflammation and immune dysregulation in BSIs, aligning with existing evidence while providing new insights specific to critically ill ICU COVID-19 patients.

#### 3.3.2. Gram Typing

The comparison of cytokine levels (Table 4) between Gram-positive and Gram-negative groups revealed no statistically significant differences among the 21 cytokines analyzed (Table 5). Nevertheless, ITAC (*p* = 0.058) and IL-17A (*p* = 0.056) had *p*-values close to the threshold for statistical significance, potentially indicating trends worth further exploration.

ITAC levels were notably higher in the Gram-positive group compared to Gram-negative (median: 90.97 vs. 33.73), with an absolute difference of 57.24, a percentage difference of −169.72%, and a fold change of 0.37. Elevated ITAC levels, typically associated with T-cell and NK-cell recruitment, have been linked to severe inflammatory states, including Gram-positive infections [70]. Although ITAC has been proposed as a potential marker for COVID-19 severity, in this study, it was primarily analyzed for its discriminatory capacity in Gram typing, as all patients were ICU COVID-19 cases [67].

Similarly, IL-17A exhibited higher levels in Gram-positive patients (median: 26.60 vs. 23.93) with a fold change of 0.90. IL-17A, associated with immune defense against Gram-positive pathogens, has been shown to influence susceptibility to Gram-positive infections and severe sepsis outcomes through genetic variations [71]. The trends observed in this cohort suggest IL-17A’s specific role in shaping immune responses to Gram-positive infections, even though the differences here did not reach statistical significance.

IL-8 levels, in contrast, were elevated in the Gram-positive group (median: 30.58 vs. 13.45 pg/mL in Gram-negative), showing the largest fold change among cytokines (2.27). Elevated IL-8 levels have been linked to neutrophil activation and heightened inflammatory responses in Gram-negative infections [72], which may reflect distinct inflammatory pathways during such infections [73].

It is important to note that the Gram-positive group had more than twice the number of patients (*n* = 15) compared to the Gram-negative group (*n* = 7), which may limit the statistical power to detect significant differences. Additionally, demographic differences, comorbidities, or variations in treatment regimens may have influenced cytokine levels. Gram-negative infections are generally associated with lipopolysaccharide-driven cytokine release, inducing higher levels of IL-6, IL-8, and IL-10, while Gram-positive infections are linked to cytokines such as IL-17A and ITAC, due to exotoxin-driven inflammatory responses [70,74].

### 3.4. Multivariate Cytokines Analysis

Despite the increasing interest in cytokine profiling for diagnostic purposes [75], the current literature focuses predominantly on traditional laboratory markers, leaving multivariate or machine learning frameworks underexplored. This section explores the potential of cytokine-based profiling for distinguishing between BSI and Gram type groups in ICU COVID-19 patients. The analysis incorporates supervised machine learning models and advanced feature selection to evaluate individual cytokines and programmatically generated cytokine ratios. Section 3.4.1 focuses on the performance metrics derived from 21 cytokines, while 3.4.2 assesses the diagnostic power of 19,250 features, including the original cytokines and 19,229 biologically relevant complex ratios. The findings emphasize the added value of feature selection and the inclusion of computationally generated ratios in improving classification accuracy and data clustering.

#### 3.4.1. Individual Cytokine Analysis

In this analysis, 21 cytokines were evaluated as features for distinguishing BSI from non-BSI patients and Gram-negative from Gram-positive infections. Supervised machine learning models were applied, and their performance was assessed using AUC, sensitivity, and specificity metrics. Sensitivity was prioritized as the most critical metric, followed by specificity and then AUC. The results are summarized in Table 6.

For BSI classification, the naïve Bayes model achieved the best overall performance on the FCBF-ranked dataset, with a sensitivity of 68.2%, specificity of 52.2%, and AUC of 0.575. This represented a modest improvement compared to the unfiltered dataset, where a random forest model achieved the highest sensitivity (59.1%) but with slightly lower specificity (52.2%) and AUC (0.505). These relatively poor results are aligned with the commonly reported results of other studies, in which, by using IL-2, IL-4, and IL-17A, AUC metrics of 0.576, 0.513, and 0.561 were obtained [76].

**Figure 3:**
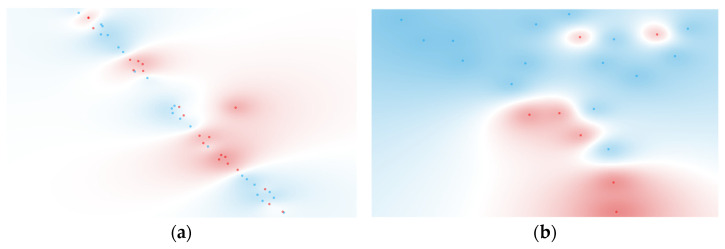
t-SNE of (**a**) 2 ranked cytokines for BSI classification (BSI: red, non-BSI: blue) and (**b**) 2 ranked cytokines for Gram typing (negative: red, positive: blue). Features were selected using the FCBF scoring method.

The sensitivity value increase from the unranked to the ranked workflow for naïve Bayes was approximately 15.4%, highlighting the contribution of feature selection in refining the dataset. FCBF ranking identified TNF-α and GM-CSF as the most relevant cytokines for BSI classification (Figure 3a), aligning with their roles in inflammatory responses during bloodstream infections. This increase, however, did fall short of a comparable result in the study, where, when used in conjunction with IL-2, IL-4, and IL-17A, the AUC jumped to a respectable 0.804 [76].

For Gram type classification, the decision tree model provided the best results for both unfiltered and FCBF-ranked workflows. On the unfiltered dataset, the model achieved a sensitivity of 71.4%, a specificity of 86.7%, and an AUC of 0.733. The FCBF-ranked workflow slightly improved the specificity (80.0%) while maintaining the same sensitivity, resulting in a marginally higher AUC of 0.750. The differences in metrics between ranked and unranked workflows were minimal, suggesting that the original cytokine dataset already captured the key discriminative information for this target group. FCBF ranking identified Fractalkine and IL-23 (Figure 3b) as the top-ranked cytokines, which is consistent with their known biological roles in inflammatory events [77,78].

These results suggest that Gram typing may be inherently easier to discriminate between than bloodstream infections, as reflected in other studies. For example, one study demonstrates that while certain cytokines, such as IFN-γ and IL-12, showed limited discriminative power (AUCs of 0.528 and 0.632, respectively), others, such as IL-8 and TNF-α, achieved highly promising results, specifically for Gram-negative bacteremia, with AUCs of 0.999 and 0.912, a sensitivity of 97.6% and 90.2%, and specificity of 100% and 87.5%, respectively [79]. Notably, the cytokines that were identified as most relevant in that study differ from those identified in the present analysis, namely, Fractalkine and IL-23, which showed strong performance for Gram typing, including Gram-negative infections. This variation may be attributed to differences in demographics and study populations; although both studies focus on ICU patients, the current study does not specifically address sepsis or mortality, in contrast to the other study. Such contextual differences likely contribute to variations in cytokine profiles and their diagnostic performance.

Regarding the t-SNE visualizations for the FCBF-ranked datasets, shown in Figure 3, these revealed moderate clustering, particularly for Gram-negative samples. This indicates that the selected features retained by the ranking process contributed to improved discrimination, although the separation between groups remained somewhat limited.

#### 3.4.2. Computationally Generated Cytokine Ratios

For this section, the dataset was expanded to include 19,229 programmatically generated cytokine ratios alongside the original 21 cytokines, for a total of 19,250 features. These ratios were derived from biologically relevant combinations of up to a sum of three pro-inflammatory cytokines in the numerator and up to a sum of three anti-inflammatory cytokines in the denominator. The same machine learning models and evaluation metrics (AUC, sensitivity, and specificity) were applied, with FCBF ranking used to refine the datasets. The results are summarized in Table 7.

**Figure 4:**
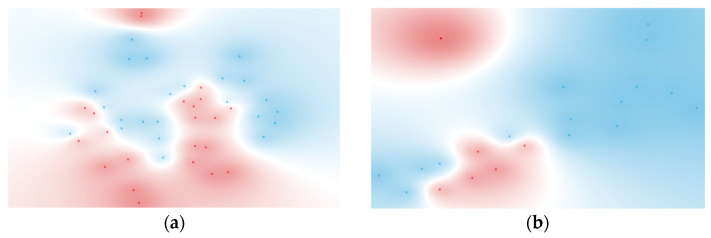
t-SNE of (**a**) six ranked cytokine ratios for BSI classification (BSI: red, non-BSI: blue) and (**b**) five ranked cytokine ratios for Gram typing (negative: red, positive: blue). Features were selected using the FCBF scoring method.

**Figure 5:**
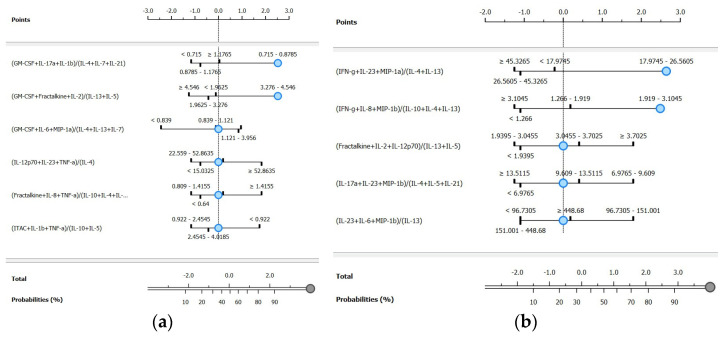
Nomograms for the ranked cytokine ratios for (**a**) BSI presence and (**b**) Gram-negative classifications.

The improvement in metrics seen in Table 7, particularly for the ranked features from BSI and Gram typing, is reflected in the t-SNE visualizations shown in Figure 4. These visualizations display distinct clusters for both BSI and Gram-negative groups, demonstrating the effectiveness of feature selection and the integration of cytokine ratios in enhancing classification and cluster separation. The specific cytokine ratios contributing to these classifications are detailed in Figure 5.

For BSI classification, the naïve Bayes model applied to the FCBF-ranked dataset achieved the highest overall performance, with a sensitivity of 90.9%, a specificity of 91.3%, and an AUC of 0.970. Compared to the unranked dataset for the same model, this represented a substantial improvement of 66.8% in sensitivity and 40.0% in specificity. The ranked workflow identified six ratios, which are visualized in the BSI nomogram (Figure 5a). Among these, the most discriminative ratio was (GM-CSF + IL-17A + IL-1β)/(IL-4 + IL-7 + IL-21), highlighting the dynamic interaction between pro-inflammatory and anti-inflammatory cytokines in identifying bloodstream infections.

These findings demonstrate superior performance metrics compared to other machine learning approaches for predicting BSI. For example, a random forest model applied to a pediatric cohort with osteoarticular infections achieved an AUC of 0.947, with a sensitivity of 84.7% and specificity of 91.7% [80]. Similarly, a multivariate logistic regression model trained on hospitalized adults with suspected BSI reported an AUC of 0.74 [81]. While these studies provide valuable insights into the application of machine learning for BSI detection, they rely on traditional laboratory parameters such as procalcitonin, neutrophil count, leukocyte count, and fever days, rather than cytokine profiling.

For Gram type classification, the naïve Bayes model again provided the best results on the FCBF-ranked dataset, with a sensitivity of 100.0%, a specificity of 80.0%, and an AUC of 0.983. This corresponds to a 40.1% increase in sensitivity and a significant improvement in specificity compared to the unranked dataset for the same model. The ranked workflow identified five ratios, visualized in the Gram-negative nomogram (Figure 5b). The most discriminative ratio was (IFN-γ + IL-23 + MIP-1α)/(IL-4 + IL-13), underscoring the relevance of these cytokines in distinguishing Gram-negative from Gram-positive infections.

The results of this analysis demonstrate higher predictive performance compared to machine learning approaches based on routine laboratory parameters, as reported in a recent study [82]. In that study, a random forest model achieved an AUC of 0.768, with a sensitivity of 75.2% and specificity of 63.79%, for distinguishing Gram-positive from Gram-negative bacteremia. Similarly, their decision tree model demonstrated an AUC of 0.679, with a sensitivity of 66% and specificity of 67.82%. These differences in performance metrics may reflect the added value of incorporating cytokine profiling into machine learning workflows, as cytokine ratios capture dynamic immune responses that are not reflected in routine laboratory parameters.

The nomograms for the ranked features of both target classes (BSI and Gram type) are shown in Figure 5. These nomograms use log odds ratios to quantify the contribution of each ranked cytokine ratio to the likelihood of correctly predicting the target class, with default positions reflecting the baseline values determined by the underlying classifier [46].

For the BSI target class, six ranked ratios were included. When the first ratio falls within the interval of 0.715–0.879, the probability of correctly identifying BSI samples is 92%, contributing 2.53 log odds ratio points. Incorporating the second ratio within the interval of 3.276–4.546 marginally increases the probability to 95%, with a total contribution of 3 log odds ratio points. The remaining four ratios, positioned at their default levels, contributed minimally to further classification probability. To the best of our knowledge, no studies to date have explored nomogram-based predictions for BSIs using complex cytokine ratios. However, in other contexts, simpler ratios or individual cytokines have been utilized. For example, a model predicting viral versus *Mycoplasma pneumoniae* infection based on the TNF-α/IL-10 ratio and other factors achieved an AUC of 0.878 [83]. Similarly, a prognostic nomogram for multiple myeloma, incorporating cytokines such as IL-6 and MIP-1α, reached a concordance index of 0.80 for survival prediction [84].

In the Gram-negative nomogram, five ranked ratios are presented. When the first ratio falls within the interval of 17.974–26.560, the probability of correct classification is 88%, contributing 2.64 log odds ratio points. Incorporating the second ratio, within the interval of 1.919–3.105, significantly increases the probability to 96%, with a total contribution of 4 log odds ratio points. This greater gain, compared to BSI classification, highlights the importance of the top-ranked ratios in Gram-negative discrimination. Existing studies have explored nomogram-based predictions for Gram-negative infections. For example, Zhang et al. [85] developed a model based on plasma IL-6 and clinical variables such as procalcitonin and C-reactive protein, achieving an AUC of 0.761, with sensitivities and specificities of 69.2% and 75.6%, respectively. Chen et al. [86] constructed a nomogram for predicting Gram-negative infections in peritoneal dialysis-associated peritonitis patients, achieving an AUC of 0.821. While these models demonstrate strong performance, they rely on routine laboratory parameters or specific clinical settings, rather than on cytokine profiling.

Overall, these nomograms provide an intuitive visualization of these differences, emphasizing the varying dependencies on key features for accurate classification. By simplifying the interpretation of complex data into clear graphical outputs, these nomograms may enable clinicians to monitor changes in cytokine ratios and adapt therapeutic strategies without engaging with the underlying mathematical or statistical principles.

### 3.5. Key Considerations and Future Directions

**Data Quality versus Quantity**

A common misconception in machine learning applications is that larger sample sizes inherently lead to better predictive accuracy. However, research has demonstrated that increasing the data volume without addressing biases and inconsistencies can amplify errors rather than improve model performance [87]. In clinical research, particularly in ICU settings, sample sizes are inherently smaller due to the nature of critically ill patient populations. This study prioritized methodological rigor and biologically meaningful feature selection to maximize the reliability of findings despite these constraints. While future studies should focus on expanding cohorts, data quality should also and always remain the primary concern.

**Importance of Data Cleaning and Normalization**

The preprocessing of medical data plays a fundamental role in model accuracy and reliability. Studies emphasize that rigorous data cleaning, normalization, and feature scaling are often more impactful in model performance than raw sample size expansion [88]. Ensuring that the cytokine data is well-curated and normalized prevents artificial variability from dominating the learning process. In this study, feature selection via FCBF was applied to reduce redundancy and improve the detection of relevant features, reinforcing the importance of preprocessing in medical-oriented applications.

**Selecting the Right Evaluation Metrics**

Another critical factor influencing the reliability of machine learning models is the selection of appropriate evaluation metrics. Relying solely on data volume without considering metric suitability can obscure underlying biases. Metrics such as accuracy can be misleading, particularly in class-imbalanced biomedical datasets, where high overall accuracy may mask poor performance in underrepresented groups. Instead, studies emphasize the importance of comprehensive evaluation using AUROC, sensitivity/specificity, and calibration curves to ensure robust model assessment [89]. Neglecting these considerations can lead to biased predictions, undermining the reliability of machine learning in medical diagnosis and therapeutic decision-making. Ensuring rigorous evaluation across all stages of model development is essential to prevent disparities and support equitable patient outcomes [90,91].

**No Sample Size is Big Enough for Universal Generalization**

Even with the most extensive multi-center datasets, true global generalization remains unattainable. Studies have shown that even large-scale machine-learning models trained on hundreds of thousands of ICU cases can experience significant performance drops when tested on external hospital datasets [92]. Similarly, an artificial intelligence-based pneumonia classifier, trained on over 158,000 chest radiographs, exhibited a substantial decrease in performance when tested on a new hospital’s population [93]. These findings reinforce that no dataset—regardless of size—can fully generalize across different demographics, hospital settings, or geographic regions. Instead of pursuing unattainable global models, future efforts should focus on developing adaptable and reproducible methodologies.

**The Path Forward: Reproducibility, Scalability, and Economic Considerations**

Machine learning-driven biomarker research should prioritize methodological validation, reproducibility, and scalability before considering economic feasibility. Demonstrating stable and reproducible results is the first step toward attracting industry collaboration and reducing costs. Cytokine assays, for instance, are traditionally costly, but by validating specific cytokine ratios as reliable biomarkers, future research can streamline testing to a select few key markers, significantly lowering costs. More importantly, the long-term objective, and a reflection of this study and its research group, is to develop a workflow that enables institutions to generate hospital or region-specific models tailored to their own patient populations. This approach circumvents the challenge of achieving universal generalization by empowering local researchers to apply the same methodology in different clinical settings, whether ICU-based or broader infirmary-level patient cohorts.

## 4. Conclusions

This study highlights the potential of cytokine profiling, complemented by machine learning, to address diagnostic challenges in ICU COVID-19 patients. By leveraging serum cytokine ratios, this study demonstrated high levels of accuracy in distinguishing BSI presence and Gram type stratification, underscoring the utility of this approach in critical care diagnostics. These findings validate cytokine ratios as meaningful biomarkers and advocate for their integration into routine diagnostic workflows. However, this study is not without its limitations. It is constrained by its sample size and reliance on a single cohort from a hospital ICU—a reflection of the inherent challenges of conducting research in critically ill populations. Additionally, the complexity and cost of comprehensive cytokine assays impose further constraints on large-scale data collection. Despite these limitations, the study, designed as a proof-of-concept approach, has showcased promising results that warrant further investigation. Future research should focus on expanding the cohort through multi-center collaborations incorporating larger, independent datasets, and integrating longitudinal analyses to improve model generalizability and external validation. Equally important is the need to ensure that predictive models remain interpretable and practical for real-world application in clinical settings, reinforcing the role of continued collaboration between research teams and healthcare professionals. These efforts aim to refine the predictive models further, enhance their clinical applicability, and ultimately optimize ICU diagnostics and patient outcomes.

## Figures and Tables

**Table 1:** Demographic and clinical characteristics of 45 ICU patients, 22 of whom had bloodstream infections confirmed by a positive hemoculture. The significance level for statistical analysis comparing the two groups was set to α = 0.01 (1%).

		No Bloodstream Infections (*n* = 23)	Bloodstream Infections (*n* = 22)	*p*-Value
Age (years), median (IQR)		58 (12)	62 (22)	0.641 #
Gender, *n* (%)	Female	5 (0.22)	3 (0.14)	0.699 +
	Male	18 (0.78)	19 (0.86)	
ECMO, *n* (%)	No	17 (0.74)	16 (0.73)	1.000 *
	Yes	6 (0.26)	6 (0.27)	
IMV, *n* (%)	No	6 (0.26)	0 (0.00)	0.022 +
	Yes	17 (0.74)	22 (1.00)	
Presence of comorbidities, *n* (%)	No	2 (0.09)	1 (0.05)	1.000 +
	Yes	21 (0.91)	21 (0.95)	
BMI, median (IQR)		29 (8)	27 (6)	0.716 #

Statistical tests used: # Mann–Whitney U, * Chi-square (χ^2^), + Fisher’s exact test.

**Table 2:** Comparison of traditional laboratory markers, cytokines, and simple ratios for bloodstream infections and Gram typing. Each feature is represented by its *p*-value and the group in which the elevated levels were observed (considering the median).

Feature	BSIs: Presence vs. Absence ^1^		Gram: Negative vs. Positive ^1^	
	*p*-Value	Elevated in ^2^	*p*-Value	Elevated in ^2^
Traditional Laboratory Markers ^3^				
CRP (mg/dL)	0.799 (ns) ^4^	BSIs	0.006 (**)	Gram+
PCT (ng/mL)	0.508 (ns)	No BSIs	0.953 (ns)	Gram−
Lymphocytes (×10^9^/L)	0.839 (ns)	No BSIs	0.014 (*)	Gram−
Neutrophil % (NE%)	0.383 (ns)	No BSIs	0.179 (ns)	Gram+
Cytokines				
IFN-γ	0.895 (ns)	BSIs	0.987 (ns)	Gram+
IL-10	0.764 (ns)	No BSIs	0.579 (ns)	Gram+
IL-17A	0.860 (ns)	No BSIs	0.056 (ns)	Gram+
IL-1β	0.900 (ns)	BSIs	0.235 (ns)	Gram+
IL-2	0.209 (ns)	No BSIs	0.943 (ns)	Gram−
IL-6	0.020 (*)	BSIs	0.553 (ns)	Gram+
IL-8	0.173 (ns)	BSIs	0.391 (ns)	Gram−
TNF-α	0.375 (ns)	BSIs	1.000 (ns)	Gram−
Simple Ratios				
IL-2/IL-10	0.920 (ns)	None ^5^	0.758 (ns)	Gram−
IL-6/IL-10	0.000 (***)	BSIs	0.888 (ns)	Gram+
IL-10/TNF-α	0.244 (ns)	No BSIs	0.564 (ns)	Gram+

^1^ No BSIs: 23 patients; BSIs: 22 patients; Gram-negative: 7 patients; Gram-positive: 15 patients. ^2^ when comparing median values between groups (with 2 decimal points). ^3^ Percentage of missing values for the traditional laboratory markers: BSIs = 12.5%; No BSIs = 82.6%; Gram-negative = 28.6%; Gram-positive = 5%). ^4^ ns: non-significant (*p* ≥ 0.05); *: *p* < 0.05; **: *p* < 0.01; ***: *p* < 0.001. ^5^ When rounding up to 2 decimal places: BSIs = 0.11 (0.110); No BSIs = 0.11 (0.108).

**Table 3:** Comparison of cytokine levels between BSI and non-BSI ICU COVID-19 patients. The table reports median values with interquartile ranges (IQR), absolute differences, percentage differences, and fold changes to highlight key disparities.

Cytokine	BSI Median (IQR)	Non-BSI Median (IQR)	Abs. Diff.	% Diff.	Fold Change
ITAC	59.42 (76.78)	106.36 (121.28)	46.93	−78.98	0.56
GM-CSF	7.42 (3.91)	8.62 (6.49)	1.21	−13.99	0.86
Fractalkine	15.52 (8.71)	16.30 (6.82)	0.77	−4.75	0.95
IFN-γ	31.01 (16.41)	28.77 (21.94)	2.24	7.79	1.08
IL-10	24.92 (59.17)	32.99 (18.45)	8.06	−24.45	0.76
MIP-3α	14.34 (10.69)	13.19 (8.04)	1.15	8.72	1.09
IL-12p70	5.64 (3.60)	4.76 (3.04)	0.88	18.56	1.19
IL-13	2.44 (1.50)	3.05 (3.17)	0.61	−19.90	0.80
IL-17A	25.50 (15.71)	29.71 (20.33)	4.20	−14.15	0.86
IL-1β	1.22 (0.52)	1.08 (0.67)	0.14	12.64	1.13
IL-2	3.31 (1.40)	3.40 (2.87)	0.09	−2.64	0.97
IL-21	10.55 (6.43)	10.29 (6.71)	0.26	2.49	1.02
IL-4	8.31 (7.53)	12.75 (7.90)	4.44	−34.81	0.65
IL-23	232.51 (125.91)	211.53 (134.44)	20.98	9.92	1.10
IL-5	6.16 (4.01)	6.16 (6.44)	0.00	0.02	1.00
IL-6	21.50 (192.18)	2.78 (10.84)	18.71	672.65	7.73
IL-7	17.41 (5.57)	17.60 (7.41)	0.19	−1.10	0.99
IL-8	16.74 (36.74)	9.65 (12.54)	7.09	73.52	1.74
MIP-1α	19.00 (11.97)	15.09 (11.92)	3.91	25.91	1.26
MIP-1β	9.49 (22.53)	16.58 (15.05)	7.09	−42.75	0.57
TNF-α	9.19 (11.55)	9.03 (4.26)	0.16	1.83	1.02

**Table 4:** Comparison of cytokine levels between Gram-negative and Gram-positive ICU COVID-19 patients. The table reports median values with interquartile ranges (IQR), absolute differences, percentage differences, and fold changes to highlight key disparities.

Cytokine	Gram− Median (IQR)	Gram+ Median (IQR)	Abs. Diff.	% Diff.	Fold Change
ITAC	33.73 (39.99)	90.97 (75.69)	57.24	−169.72	0.37
GM-CSF	7.32 (2.37)	7.51 (4.01)	0.19	−2.58	0.97
Fractalkine	23.25 (19.38)	13.38 (4.80)	9.86	42.43	1.74
IFN-γ	28.98 (22.68)	33.03 (15.15)	4.05	−13.96	0.88
IL-10	21.88 (245.68)	25.87 (53.37)	3.99	−18.21	0.85
MIP-3α	9.60 (34.20)	14.84 (9.90)	5.24	−54.51	0.65
IL-12p70	4.74 (2.86)	5.85 (3.25)	1.11	−23.44	0.81
IL-13	2.29 (1.11)	3.33 (1.57)	1.05	−45.73	0.69
IL-17A	23.93 (8.84)	26.60 (15.79)	2.67	−11.16	0.90
IL-1β	1.05 (0.51)	1.27 (0.49)	0.21	−20.23	0.83
IL-2	3.48 (1.66)	3.22 (1.25)	0.25	7.28	1.08
IL-21	11.68 (4.47)	9.48 (6.21)	2.21	18.89	1.23
IL-4	11.60 (8.22)	6.35 (7.46)	5.25	45.24	1.83
IL-23	223.29 (110.93)	249.41 (186.69)	26.12	−11.70	0.90
IL-5	5.59 (4.07)	7.21 (6.65)	1.62	−28.91	0.78
IL-6	3.97 (12207.22)	21.55 (157.65)	17.58	−442.75	0.18
IL-7	14.47 (4.08)	18.26 (6.09)	3.79	−26.19	0.79
IL-8	30.58 (642.58)	13.45 (27.51)	17.12	56.00	2.27
MIP-1α	17.22 (5.03)	19.99 (20.05)	2.76	−16.05	0.86
MIP-1β	8.24 (12.85)	10.74 (27.35)	2.51	−30.45	0.77
TNF-α	13.60 (16.65)	8.72 (10.74)	4.89	35.92	1.56

**Table 5:** *p*-values for the comparison between Gram-negative and Gram-positive ICU COVID-19 patients for the 21 cytokines analyzed.

Cytokine	*p*-Value
ITAC	0.058 (ns) ^1^
GM-CSF	0.704 (ns)
Fractalkine	0.147 (ns)
IFN-γ	0.987 (ns)
IL-10	0.579 (ns)
MIP-3α	0.433 (ns)
IL-12p70	0.182 (ns)
IL-13	0.797 (ns)
IL-17A	0.056 (ns)
IL-1β	0.235 (ns)
IL-2	0.943 (ns)
IL-21	0.665 (ns)
IL-4	0.782 (ns)
IL-23	0.159 (ns)
IL-5	0.485 (ns)
IL-6	0.553 (ns)
IL-7	0.119 (ns)
IL-8	0.391 (ns)
MIP-1α	0.633 (ns)
MIP-1β	0.430 (ns)
TNF-α	1.000 (ns)

^1^ ns: non-significant.

**Table 6:** Performance of five-fold cross-validation for distinguishing between BSI and gram type in ICU COVID-19 patients, using 21 cytokines. The metrics presented correspond to BSI presence and Gram-negative cases, obtained using various machine learning models, before and after applying FCBF for feature selection.

Target Group	Model	AUC	Sensitivity	Specificity
BSI	kNN	0.625	0.409	0.739
	Naïve Bayes	0.620	0.455	0.652
	Random Forest	0.505	0.591	0.522
	SVM	0.690	0.273	0.478
	Decision Tree	0.520	0.500	0.565
BSI Ranked: FCBF	kNN	0.525	0.409	0.739
	Naïve Bayes	0.575	0.682	0.522
	Random Forest	0.610	0.545	0.522
	SVM	0.510	0.227	0.652
	Decision Tree	0.430	0.409	0.478
Gram	kNN	0.517	0.143	0.667
	Naïve Bayes	0.533	0.571	0.533
	Random Forest	0.567	0.286	0.667
	SVM	0.633	0.000	1.000
	Decision Tree	0.733	0.714	0.867
Gram Ranked: FCBF	kNN	0.483	0.143	0.933
	Naïve Bayes	0.700	0.571	0.667
	Random Forest	0.733	0.286	0.867
	SVM	0.300	0.000	1.000
	Decision Tree	0.750	0.714	0.800

**Table 7:** Performance of five-fold cross-validation for distinguishing BSI and Gram type in ICU COVID-19 patients, using 21 cytokines and 19,229 ratios. The metrics presented correspond to BSI presence and Gram-negative cases, obtained using various machine learning models before and after applying FCBF for feature selection.

Target Group	Model	AUC	Sensitivity	Specificity
BSI	kNN	0.635	0.455	0.783
	Naïve Bayes	n.a. ^1^	0.545	0.652
	Random Forest	0.625	0.500	0.696
	SVM	0.530	0.455	0.739
	Decision Tree	0.675	0.727	0.565
BSI Ranked: FCBF	kNN	0.665	0.636	0.652
	Naïve Bayes	0.970	0.909	0.913
	Random Forest	0.895	0.682	0.870
	SVM	0.520	0.500	0.652
	Decision Tree	0.830	0.727	0.870
Gram	kNN	0.517	0.143	0.800
	Naïve Bayes	n.a. ^1^	0.714	0.133
	Random Forest	0.600	0.286	0.800
	SVM	0.533	0.000	1.000
	Decision Tree	0.567	0.429	0.733
Gram Ranked: FCBF	kNN	0.800	0.714	0.667
	Naïve Bayes	0.983	1.000	0.800
	Random Forest	0.900	0.857	0.867
	SVM	0.667	0.000	0.867
	Decision Tree	0.800	0.714	0.867

^1^ Some scores could not be computed (n.a.—not available)

## Data Availability

The data presented in this study are not publicly available, due to privacy or ethical restrictions, and due to the ongoing nature of the study.
